# The prognostic significance of monoclonal immunoglobulin gene rearrangement in conjunction with histologic B‐cell aggregates in the bone marrow of patients with diffuse large B‐cell lymphoma

**DOI:** 10.1002/cam4.679

**Published:** 2016-02-29

**Authors:** Yoon Ah Cho, Woo Ick Yang, Jae‐Woo Song, Yoo Hong Min, Sun Och Yoon

**Affiliations:** ^1^ Department of PathologyYonsei University College of MedicineSeoulKorea; ^2^ Department of Laboratory MedicineYonsei University College of MedicineSeoulKorea; ^3^ Department of Internal MedicineYonsei University College of MedicineSeoulKorea

**Keywords:** Bone marrow, diffuse large B‐cell lymphoma, immunoglobulin genes, lymphoid aggregates, prognosis

## Abstract

Bone marrow involvement (BMI) is a well‐known poor prognostic factor in patients with diffuse large B‐cell lymphoma (DLBCL). This study robustly investigated the significance of monoclonal immunoglobulin gene rearrangement combined with histologic B‐cell aggregates in bone marrow (BM) in the detection of a poor prognostic group. Pretreatment BM samples of 394 DLBCL patients were analyzed via the immunoglobulin gene rearrangement study and the microscopic examination. Monoclonal immunoglobulin gene rearrangement was detected in 25.4% of cases. Histologic B‐cell aggregates with the features of large B‐cell lymphoma aggregates, small cell B‐cell lymphoma aggregates, or B‐cell aggregates of unknown biological potential were observed in 12% of cases (6.9%, 1.3%, and 3.8%, respectively). Histologic B‐cell aggregates were more associated with monoclonality than polyclonality. Cases with both monoclonality and histologic B‐cell aggregates demonstrated close association with poor prognostic factors such as a higher International Prognostic Index score and showed an inferior overall survival rate when compared to cases with only monoclonality or only histologic B‐cell aggregates. From the findings, a combination of monoclonality and histologic B‐cell aggregates within the bone marrow was highly associated with poor prognosis and could be used to determine high‐risk DLBLC patients with greater sensitivity and specificity than conventional microscopic examination or immunoglobulin gene rearrangement study alone.

## Introduction

In patients with diffuse large B‐cell lymphoma (DLBCL), the most common subtype of malignant lymphoma worldwide, bone marrow involvement (BMI) is known to be a poor prognostic factor for patient outcome. BMI is categorized as extranodal site involvement and Ann‐Arbor stage IV disease and is therefore associated with the International Prognostic Index (IPI), the most reliable prognostic indicator of lymphoma patients 1, 2, 3. Even in the rituximab era, BMI remains a poor prognostic factor for DLBCL patients 4, 5. In published studies, approximately 10% of DLBCL cases exhibited bone marrow involvement at the time of diagnosis 2, 3, 4, 5.

Bone marrow biopsies and aspirations sampled from the iliac crest bone, which are the conventional means of BMI detection, are notorious for their high false‐negative rates 2, 3, 5. To overcome this limitation, the usefulness of ancillary tests, including immunoglobulin gene rearrangement study, flow cytometry and immunohistochemistry, have been evaluated. Previous studies show that these ancillary tests can improve the detection rate of BMI and predict the outcome of DLBCL patients. Among them, detection of monoclonal immunoglobulin gene rearrangement has been considered as an alternative tool for detecting minimal tumor cells 6, 7. However, the implications of detecting monoclonal immunoglobulin gene rearrangement in conjunction with histologic B‐cell aggregates have not been robustly studied in terms of patient prognosis. In this study, the clinical value of monoclonal immunoglobulin gene rearrangement in conjunction with histologic B‐cell aggregates was investigated in a large number of DLBCL patients.

### Methods

## Patients and samples

Pretreatment bone marrow samples of patients with diffuse large B‐cell lymphoma (DLBCL) were analyzed for this study. Patients were diagnosed with DLBCL according to the World Health Organization classification criteria 1 at Severance Hospital from July 2010 to March 2015. Among the total number of patients diagnosed as DLBCL, 394 cases were selected for this study according to the inclusion criteria: results of both the immunoglobulin gene rearrangement study and the histologic assessment were available based on the quality of the bone marrow specimen. Information on clinicopathologic factors, treatment, and survival data were obtained from the medical records. Systemic chemotherapy was performed in 92% of cases (363 of 394), and 74% of the patients (292 of 394) were treated with rituximab. The Institutional Review Board approved this study.

### Histologic assessment of bone marrow involvement and immunoglobulin gene rearrangement study

The trephine bone marrow biopsy specimens were sampled for lymphoma staging before therapy. The histology of each trephine bone marrow biopsy was reviewed by hematopathologists. Sample adequacy was evaluated grossly and microscopically. The formalin‐fixed paraffin‐embedded (FFPE) tissue specimens of the bone marrow biopsy were stained with H&E, and immunohistochemical analysis of B‐cell markers (CD20, CD79a, and/or PAX5) was performed to detect B‐cell proliferation. Bone marrow involvement was defined according to histologic criteria and immunohistochemistry of B‐cell markers 3, 4.

The histologic features of B‐cell infiltration within bone marrow were defined as large B‐cell lymphoma aggregates, small cell B‐cell lymphoma aggregates, or B‐cell aggregates of unknown biological potential. Proliferations of large and/or pleomorphic B cells with various patterns of paratrabecular, interstitial, and/or diffuse growth were defined as large B‐cell lymphoma aggregates 3, 4 (Fig. S1A and B). Proliferations of small‐ to medium‐sized mature B cells that occupied large proportions of marrow surfaces and showed characteristic growth patterns indicative of malignant lymphoid aggregates such as paratrabecular location, infiltrative edges, inclusion of fat cells or location surrounding large sinuses 8, 9 were defined as small cell B‐cell lymphoma aggregates (Fig. S1C and D). Sparse collections of nonparatrabecular, interstitial aggregates of small‐ to medium‐sized mature B cells were defined as B‐cell aggregates of unknown biological potential because lymphoid aggregates of this category could not fulfill the criteria of lymphoma due to low cellularity and/or lack of histologic characteristics indicative of malignant lymphoid aggregates 8, 9 (Fig.S1E–H).

Bone marrow aspirates of the DLBCL patients were used for the detection of monoclonal gene rearrangement of immunoglobulin heavy (IgH) chain and immunoglobulin kappa (IgK) chain. PCR assays were performed using BIOMED‐2 multiplex primers according to the manufacturer's protocol (InVivoScribe, San Diego, CA, USA). Reaction assays for IgH and IgK gene rearrangement tests included the full set of five reactions targeting IGH (IGH_A_: FR1‐JH; IGH_B_: FR2‐JH; IGH_C_: FR3‐JH; IGH_D_: DH1–6‐JH; IGH_E_: DH7‐JH) and two reactions targeting IGK (IGK_A_: V_k_‐J_k_; IGK_B_: V_k_‐Kde + intron‐Kde) 10. Cases showing monoclonal immunoglobulin gene rearrangement were defined as the monoclonality group, and cases showing polyclonal immunoglobulin gene rearrangement were defined as the polyclonality group. According to clonal status of immunoglobulin gene rearrangement and histologic B‐cell aggregates, the cases were divided into the following four groups: both monoclonality and histologic B‐cell aggregates, monoclonality only (monoclonality and absence of B‐cell aggregates), histologic B‐cell aggregates only (polyclonality and histologic B‐cell aggregates) and no abnormality (polyclonality and absence of B‐cell aggregates).

### Statistical analysis

Differences between the variables examined were analyzed using Fisher's exact test. Multiple logistic regression analysis was performed to examine the relationships of multiple parameters. Variables predictive of overall survival were analyzed using uni‐ and multivariate Cox proportional hazards models. Overall survival curves were estimated using the Kaplan–Meier method and compared using the log‐rank test. Overall survival times were measured from the date of lymphoma diagnosis to the date of lymphoma‐related death or the last follow‐up visit. The average follow‐up period was 24.7 months (range, 0.5–66.0 months). A two‐sided *P* value <0.05 was considered to be statistically significant. All statistical analyses were carried out using SPSS software, version 20.0 for Windows (IBM, Armonk, NY).

## Results

### Patterns of immunoglobulin gene rearrangement and histologic features in bone marrow

**Figure 1:**
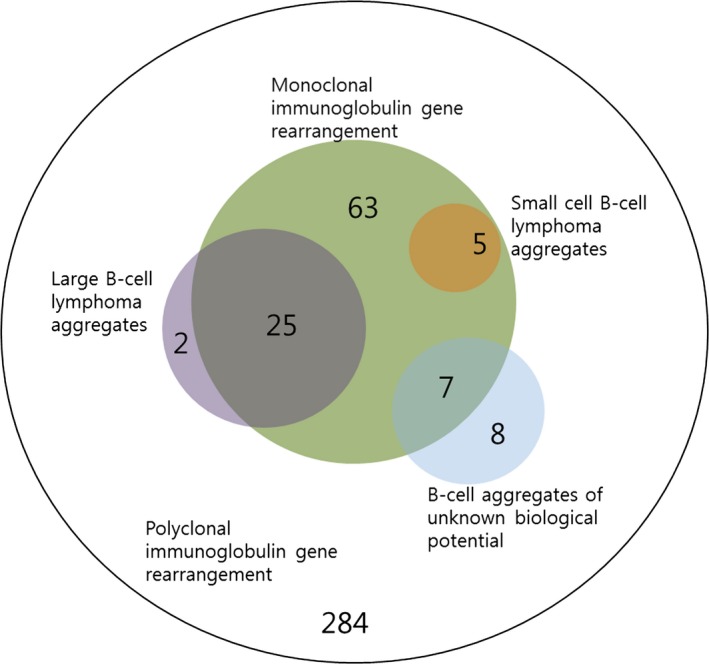
Venn diagram showing the relationships between clonal status of immunoglobulin gene rearrangement and histologic B‐cell aggregates. Among 394 cases, 100 (green) showed monoclonal immunoglobulin gene rearrangement, 27 showed bone marrow involvement of large B‐cell lymphoma aggregates (violet), five showed small cell B‐cell lymphoma aggregates (scarlet), and seven showed B‐cell aggregates of unknown biological potential (blue). The remaining 284 cases showed no abnormalities in both clonality and histologic B‐cell aggregates

The distribution of cases according to the clonal status of immunoglobulin gene rearrangement and histologic B‐cell aggregates is presented in Figure 1. In the immunoglobulin gene rearrangement study, monoclonal IgH and/or IgK gene rearrangement was observed in 25.4% of cases (*n* = 100), and 74.6% of cases (*n* = 294) showed polyclonal immunoglobulin gene rearrangement. In the histologic analysis, large B‐cell lymphoma aggregates were noted in 6.9% (*n* = 27), small cell B‐cell lymphoma aggregates in 1.3% (*n* = 5), and B‐cell aggregates of unknown biological potential in 3.8% (*n* = 15). Of the 394 cases, 88.1% (*n* = 347) did not show B‐cell aggregates. Among 100 cases showing monoclonal immunoglobulin gene rearrangement (the monoclonality group), histologic B‐cell aggregates were noted in 37% of cases (37 of 100): 25% with large B‐cell lymphoma aggregates, 5% with small cell B‐cell lymphoma aggregates, and 7% with B‐cell aggregates of unknown biological potential. Within the monoclonality group, 63% of cases (63 of 100) did not show B‐cell aggregates. Among the 294 cases showing polyclonal immunoglobulin gene rearrangement (the polyclonality group), 96.6% (*n* = 284) did not show B‐cell aggregates. The remaining cases were two (0.7%) with large B‐cell lymphoma aggregates and eight (2.7%) with B‐cell aggregates of unknown biological potential. The proportion of histologic B‐cell aggregates was higher in the monoclonality group than in the polyclonality group (37% vs. 3.4%; *P* < 0.001). Overall, 9.4% (*n* = 37) showed both monoclonality and histologic B‐cell aggregates, 16% (*n* = 63) monoclonality only, 2.5% (*n* = 10) histologic B‐cell aggregates only, and 72.1% (*n* = 284) no abnormality.

### Monoclonal immunoglobulin gene rearrangement and histologic B‐cell aggregates in association with clinicopathological factors

Overall associations of monoclonal immunoglobulin gene rearrangement and/or histologic B‐cell aggregates with clinicopathological variables were analyzed using Fisher's exact test, summarized in Table 1. Monoclonality was more associated with the previously known poor prognostic factors of age >60 years, elevated lactate dehydrogenase (LDH), Eastern Cooperative Oncology Group (ECOG) performance score ≥2, extranodal site involvement ≥2, Ann‐Arbor stage III/IV, and high IPI score than polyclonality. Histologic B‐cell aggregates were also more associated with elevated LDH, extranodal site involvement ≥2, Ann‐Arbor stage III/IV, and high IPI score than the absence of B‐cell aggregates. Germinal center B‐cell–like (GCB) type or non‐GCB type DLBCL showed no association with clonal status of immunoglobulin gene rearrangement or histologic B‐cell aggregates.

**Table 1:** Monoclonal immunoglobulin gene rearrangement and histologic B‐cell aggregates in association with clinicopathological factors

Factors	Category	Immunoglobulin gene rearrangement			Histologic B‐cell aggregates		
		Polyclonal (*n* = 284)	Monoclonal (*n* = 100)	*P* value	Absence of B‐cell aggregates (*n* = 347)	B‐cell aggregates (*n* = 47)	*P* value
Diffuse large B‐cell lymphoma (DLBCL) type	GCB	31.7%	27.6%	0.567	29.5%	40.6%	0.225
	NGC	68.3%	72.4%		70.5%	59.4%	
Age	≤60	51.7%	35.0%	0.005	49.0%	36.2%	0.120
	>60	48.3%	65.0%		51.0%	63.8%	
LDH	Not elevated	56.6%	38.9%	0.003	55.5%	27.3%	0.001
	elevated	43.4%	61.1%		44.5%	72.7%	
ECOG PS	<2	86.2%	72.9%	0.005	83.9%	75.0%	0.141
	≥2	13.8%	27.1%		16.1%	25.0%	
Extranodal site	<2	77.5%	53.1%	<0.001	75.1%	43.2%	<0.001
	≥2	22.5%	46.9%		24.9%	56.8%	
Ann‐Arbor stage	I/II	60.0%	34.4%	<0.001	58.2%	18.2%	<0.001
	III/IV	40.0%	65.6%		41.8%	81.8%	
IPI score	Low (0, 1)	48.6%	27.4%	<0.001	47.0%	14.0%	<0.001
	Intermediate (2, 3)	40.5%	45.3%		40.2%	53.5%	
	High (4, 5)	10.9%	27.4%		12.8%	32.6%	

GCB, germinal center B‐cell like; NGC, non‐GCB; LDH, lactate dehydrogenase; ECOG PS, Eastern Cooperative Oncology Group performance score; IPI, International Prognostic Index.

**Figure 2:**
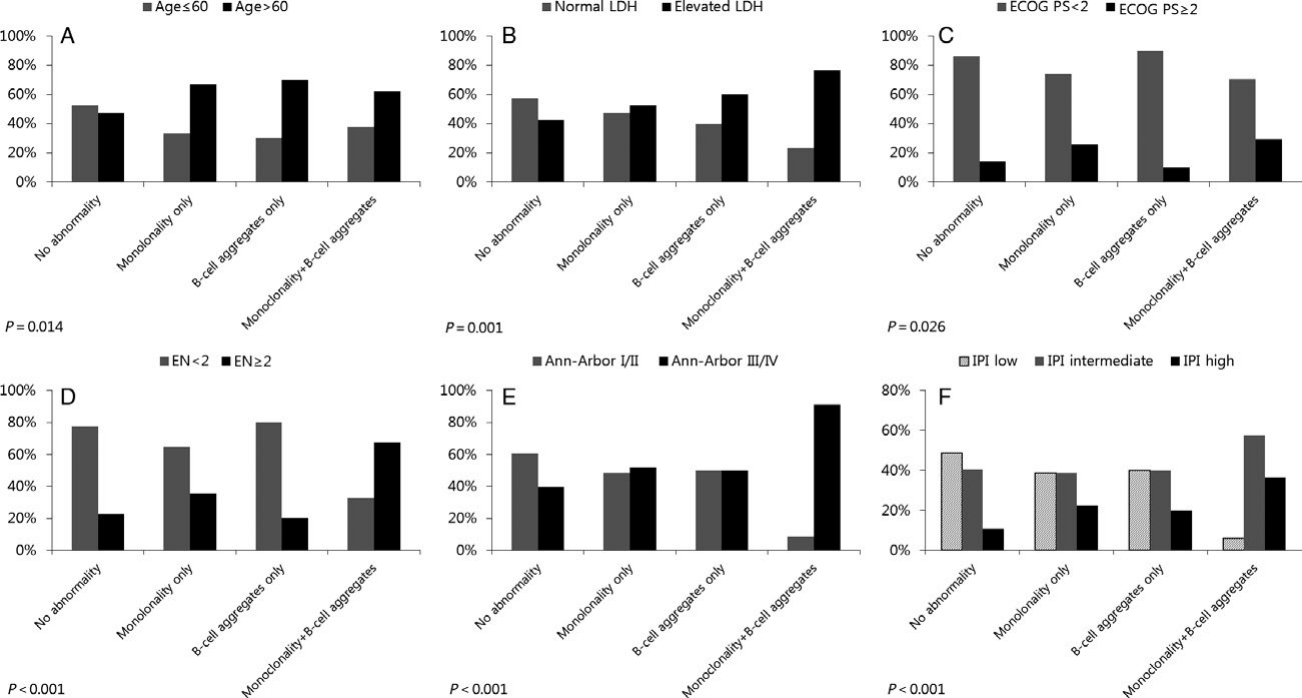
When compared to the rates of known prognostic factors (A–F) in the group with no abnormalities, the rates of age > 60 (A), elevated lactate dehydrogenase (LDH) (B), ECOG performance score ≥2 (C), extranodal site involvement ≥2 (D), Ann‐Arbor stage III/IV (E), and high IPI score (F) were higher in the three groups with monoclonality only (monoclonality and absence of B‐cell aggregates), B‐cell aggregates only (polyclonality and B‐cell aggregates), and both monoclonality and B‐cell aggregates, particularly, in the group with both monoclonality and B‐cell aggregates.

The proportion of patients with age >60, elevated LDH, ECOG performance score ≥2, extranodal site involvement ≥2, Ann‐Arbor stage III/IV, and high IPI score was higher in the groups showing monoclonality and/or B‐cell aggregates, particularly in the group showing both monoclonality and B‐cell aggregates, when compared with the group with no abnormalities (Fig. 2 A–F). On multiple logistic regression analysis, intermediate or high IPI score as well as the individual IPI risk factors except age >60 were significantly related to the group with both monoclonality and B‐cell aggregates. The factors of extranodal site involvement ≥2 and Ann‐Arbor stage III/IV as well as an intermediate or high IPI score were independently related to the group with both monoclonality and B‐cell aggregates. For the group with monoclonality only, the factors of age >60 and high IPI score were independently related. The group with B‐cell aggregates only did not demonstrate significant relations to the IPI risk factors (Table 2).

**Table 2:** Multiple logistic regression analysis

Group (vs. no abnormality)	Variables	Univariate regression analysis			Multivariate regression analysis		
		*P* value	OR	95% CI	*P* value	OR	95% CI
Monoclonality only	Age (>60 vs. ≤60)	0.007	2.2	1.2–3.9	0.020	2.1	1.1–3.8
	LDH(elevated vs. normal)	0.171	1.5	0.8–2.6	0.874	1.1	0.6–2.0
	ECOGPS(≥2 vs. <2)	0.023	2.1	1.1–4.2	0.183	1.6	0.8–3.4
	Extranodal site(≥2 vs. <2)	0.035	1.9	1.0–3.4	0.128	1.7	0.9–3.4
	Ann‐Arbor stage (III–IV vs. I–II)	0.085	1.6	0.9–2.8	0.888		0.5–2.1
	IPI scores (vs. low risk)						
	Intermediate risk	0.551	1.2	0.7–2.2			
	High risk	0.012	2.7	1.2–5.8			
B‐cell aggregates only	Age (>60 vs. ≤60)	0.177	2.6	0.7–10.2	0.208	2.5	0.6–10.1
	LDH(elevated vs. normal)	0.29	2.0	0.6–7.3	0.37	1.9	0.5–7.6
	ECOGPS(≥2 vs. <2)	0.725	0.7	0.1–5.6	0.449	0.4	0.1–3.7
	Extranodal site(≥2 vs. <2)	0.85	0.9	0.2–4.1	0.727	0.7	0.1–4.1
	Ann‐Arbor stage (III–IV vs. I–II)	0.514	1.5	0.4–5.4	0.711	1.3	0.3–5.6
	IPI scores (vs. low risk)						
	Intermediate risk	0.347	2.3	0.4–13.2			
	High risk	0.793	1.2	0.3‐4.9			
Both monoclonality and B‐cell aggregates	Age (>60 vs. ≤60)	0.098	1.8	0.9–3.7	0.555	1.3	0.6–2.8
	LDH(elevated vs. normal)	0.001	4.3	1.9–9.9	0.205	1.8	0.7–4.5
	ECOGPS(≥2 vs. <2)	0.022	2.6	1.1–5.8	0.62	1.3	0.5–3.1
	Extranodal site(≥2 vs. <2)	<0.001	7.2	3.3–15.5	0.007	3.2	1.4–7.4
	Ann‐Arbor stage (III–IV vs. I–II)	<0.001	15.7	4.7–52.7	0.003	7.1	1.9–26.2
	IPI scores (vs. low risk)						
	Intermediate risk	0.001	11.5	2.6–50.3			
	High risk	<0.001	27.7	5.9–130.6			

The analysis for IPI scores was independently performed as the factors (age, lactate dehydrogenase (LDH), ECOGPS, extranodal site involvement, Ann‐Arbor stage) included IPI risk stratification by definition.

OR, odds ratio; 95%CI, 95% confidence interval for odds ratio.

### Association with patient survival

**Figure 3:**
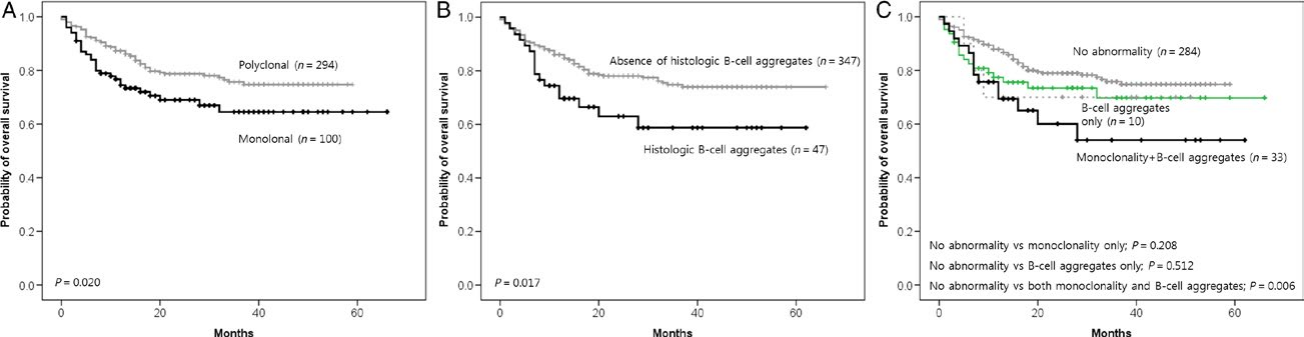
Cases showing monoclonal immunoglobulin gene rearrangement (Monoclonal) were associated with inferior overall survival (OS) rates compared to cases with polyclonal immunoglobulin gene rearrangement (Polyclonal) (A). Cases with histologic B‐cell aggregates were more highly associated with an inferior OS rate than cases showing absence of histologic B‐cell aggregates (B). When compared to cases showing no abnormalities, cases showing both monoclonality and histologic B‐cell aggregates showed an inferior OS rate (C). Cases showing monoclonality only or cases showing B‐cell aggregates only showed no difference in OS rate when compared with cases with no abnormalities (C). Survival function curves and survival rates were determined using the Kaplan–Meier method, and differences in survival rates were compared using the log‐rank test.

Cases showing monoclonal immunoglobulin gene rearrangement were associated with an inferior overall survival (OS) rate compared to cases with polyclonality (*P* = 0.020; Fig. 3A). According to histology, cases with histologic B‐cell aggregates were more associated with an inferior OS rate than cases showing absence of B‐cell aggregates (*P* = 0.017; Fig. 3B). When compared with cases showing no abnormalities (polyclonality and absence of B‐cell aggregates), cases showing both monoclonality and histologic B‐cell aggregates showed an inferior OS rate (*P* = 0.006; Fig. 3C). Cases showing monoclonality only and cases showing histologic B‐cell aggregates only showed no difference in OS compared to cases with no abnormalities (*P* = 0.208 and 0.512, respectively; Fig. 3C). On multivariate analysis for overall survival, however, monoclonality and/or B‐cell aggregates did not show statistical significance as an independent prognostic factor; only the IPI score was revealed to be an independent prognostic factor for overall survival (Table 3).

**Table 3:** Univariate and multivariate Cox proportional hazards models for overall survival

Variables	Univariate analysis			Multivariate analysis		
	*P* value	HR	95% CI	*P* value	HR	95% CI
Age (>60 vs. ≤60)	<0.001	2.9	1.8–4.6			
LDH(elevated vs. normal)	0.001	2.1	1.4–3.2			
ECOGPS(≥2 vs. <2)	<0.001	3.3	2.1–5.2			
Extranodal site(≥2 vs. <2)	0.352	1.2	0.8–1.9			
Ann‐Arbor stage (III–IV vs. I–II)	<0.001	2.3	1.5–3.5			
IPI scores (vs. low risk)						
Intermediate risk	<0.001	3.3	1.9–5.6	<0.001	3.2	1.8–5.5
High risk	<0.001	4.9	2.6–9.1	<0.001	4.6	2.4–8.6
The case group (vs. no abnormality)						
Monoclonality only	0.209	1.4	0.8–2.4	0.442	1.2	0.7–2.1
B‐cell aggregates only	0.521	1.5	0.5–4.7	0.737	1.2	0.4–3.9
Both monoclonality and B‐cell aggregates	0.009	2.2	1.2–3.9	0.458	1.3	0.7–2.4

## Discussion

This study investigated the prognostic implications of the molecular detection of monoclonal immunoglobulin gene rearrangement in conjunction with the histologic detection of B‐cell aggregates in the pretreatment bone marrow of patients with diffuse large B‐cell lymphoma (DLBCL). This study aimed to determine the prognostic implications of B‐cell aggregates accompanying monoclonality in order to improve the sensitivity and specificity of bone marrow tests for bone marrow involvement in DLBCL patients. Thus, the prognostic significance of histologic aggregates of monoclonal B cells (both monoclonality and B‐cell aggregates) was compared with that of monoclonality only (monoclonal immunoglobulin gene rearrangement and absence of B‐cell aggregates), B‐cell aggregates only (polyclonality and B‐cell aggregates), and no abnormalities (polyclonality and absence of B‐cell aggregates).

In this study, monoclonality was detected in 25.4% of cases, and monoclonality itself was closely related to poor prognostic factors such as high IPI scores and associated with inferior overall survival. In a previous study on the bone marrow staging of 155 DLBCL patients, 22.6% of cases (35 of 155) showed monoclonal immunoglobulin gene (IgH and/or IgK) rearrangement in the bone marrow aspirates and/or peripheral blood, and this monoclonality was related to inferior overall survival 6. The positivity rate of monoclonality and the close association of monoclonality with inferior OS were similar to the present findings. These findings indicate that immunoglobulin gene rearrangement studies of bone marrow can be used to detect a poor prognostic group among DLBCL patients with higher sensitivity than conventional microscopic examination; bone marrow involvement (BMI) of DLBCL, which was defined by conventional histologic criteria 3, 4, was noted in only 6.9% of tested cases in this study. Monoclonality only (monoclonal immunoglobulin gene rearrangement and absence of B‐cell aggregates), however, did not show independent association with known prognostic factors except for the factor of age >60. In addition, monoclonality only showed no relation to patient survival. These findings indicate that monoclonality without the formation of B‐cell aggregates may be related monoclonal B‐cell lymphocytosis, an asymptomatic indolent syndrome wherein lower numbers of monoclonal B cells are present in the peripheral blood, usually in late adulthood and particularly in those older than 60 years. This condition is known to rarely progress into malignant lymphoma (specifically, chronic lymphocytic leukemia), and most cases show benign clinical features 11, 12, 13. Pseudoclonality or false‐positive results might also be suggested for cases with monoclonality only; however, the BIOMED‐2 multiplex PCR method is known to detect clonal B cells with high sensitivity and specificity 14, 15.

Proliferation of large B cells with various growth patterns is defined as BMI of DLBCL based on previously reported criteria 3, 4. Although several recent studies show that small cell B‐cell lymphoma involvement cannot independently predict a poor clinical outcome 3, 4, it should be noted that the determination of B‐cell size within the bone marrow is frequently difficult, likely due to low cellularity, overflow of admixed hematopoietic precursors, or artificial atrophy during tissue processes such as decalcification and dehydration. These factors seem to be related to the high false‐negative rate for BMI. We considered the implications of formation of lymphoid aggregates, as the histologic/structural growth pattern could be more easily detected at the microscopic level. In addition, the possible limitations of microscopic examination might have been reduced, as structural changes may have been relatively less affected by artifacts such as tissue processes. Although small (<600 *μ*m) nonparatrabecular, well‐circumscribed aggregates of small‐ to medium‐sized mature B cells within bone marrow have been considered to be benign lymphoid aggregates in previous studies 8, 9, the clonal status of such B‐cell aggregates was not robustly investigated in those studies. In fact, this study noted that the growth pattern of histologic B‐cell aggregates itself was closely related to poor prognostic factors such as high IPI scores and significantly associated with an inferior OS rate. These findings suggest that the growth pattern of histologic B‐cell aggregates may have prognostic relevance. In addition, histologic B‐cell aggregates can more sensitively detect a poor prognostic group of DLBCL patients; in this study BMI by conventional definition was noted in only 6.9% of cases, whereas histologic B‐cell aggregates were noted in 12% of cases. B‐cell aggregates only (polyclonality and histologic B‐cell aggregates), however, did not show independent association with the known poor prognostic factors or patient survival. In this study, most (80%, 2 of 10; Fig.1) cases showing B‐cell aggregates only had histologic types of unknown biological potential. These present findings imply that polyclonal B‐cell aggregates, particularly histologic types of unknown biological potential, could be considered as benign lymphoid aggregates. In other words, B‐cell aggregates of unknown biological potential could be determined to be of benign or malignant biologic potential through the immunoglobulin gene rearrangement study.

To overcome the possible limitation of immunoglobulin gene rearrangement study or conventional microscopic histologic examination as a single test modality for BMI, we focused on the combination of monoclonality and histologic B‐cell aggregates (i.e., monoclonal B‐cell aggregates). In terms of prognosis, patients with monoclonality in conjunction with histologic B‐cell aggregates were determined to be the highest risk group. Poor prognostic factors such as high IPI scores were significantly related to cases with monoclonal B‐cell aggregates (both monoclonality and B‐cell aggregates). In addition, cases with both monoclonality and B‐cell aggregates showed an inferior OS rate compared to cases with no abnormalities. Cases with monoclonality only or B‐cell aggregates only may be in the same risk group as cases showing no abnormalities in terms of DLBCL prognosis.

This study had several limitations. Although the monoclonal B‐cell aggregates (both monoclonality and B‐cell aggregates) revealed poor patient survival on univariate analysis, the factor was not determined to be an independent prognostic factor on multivariate analysis. In particular, the observation time was relatively short. A longer time observation is necessary to assess the long‐term effects of monoclonal B‐cell aggregates in terms of patient prognosis.

In conclusion, histologic B‐cell aggregates were found to be significantly associated with monoclonality. Monoclonality combined with histologic B‐cell aggregates within the bone marrow was most highly associated with poor prognosis and could be used to detect high‐risk DLBLC patients with greater sensitivity and specificity than conventional microscopic examination only or immunoglobulin gene rearrangement study only.

## Conflicts of Interest

None declared.

## Supporting information


**Figure S**1. (A) A representative case of the involvement of large B‐cell lymphoma (DLBCL) that shows diffuse proliferation of large B cells (A and B). A representative case of involvement of small cell B‐cell lymphoma that shows small‐ to medium‐sized low‐grade mature B cells in patterns of large aggregates of infiltrative edges, occupying large proportions of marrow surfaces (C and D). Representative cases of small nonparatrabecular, interstitial aggregates of small‐ to medium‐sized mature B cells, which did not fulfill the criteria of lymphoma due to low cellularity of the proliferating B cells or lack of histologic characteristics for malignant lymphoid aggregates (E–F and G–H). Figures of H&E staining and CD20 immunostaining were captured at ×200 magnification. Figures of insets show more details of tumor cells.
