# Supplementary figures and images for: The prognostic significance of monoclonal immunoglobulin gene rearrangement in conjunction with histologic B‐cell aggregates in the bone marrow of patients with diffuse large B‐cell lymphoma
Source: Cancer Med. 2016 Feb 29;5(6):1066–73. doi: 10.1002/cam4.679 (PMC4924364; doi:10.1002/cam4.679)

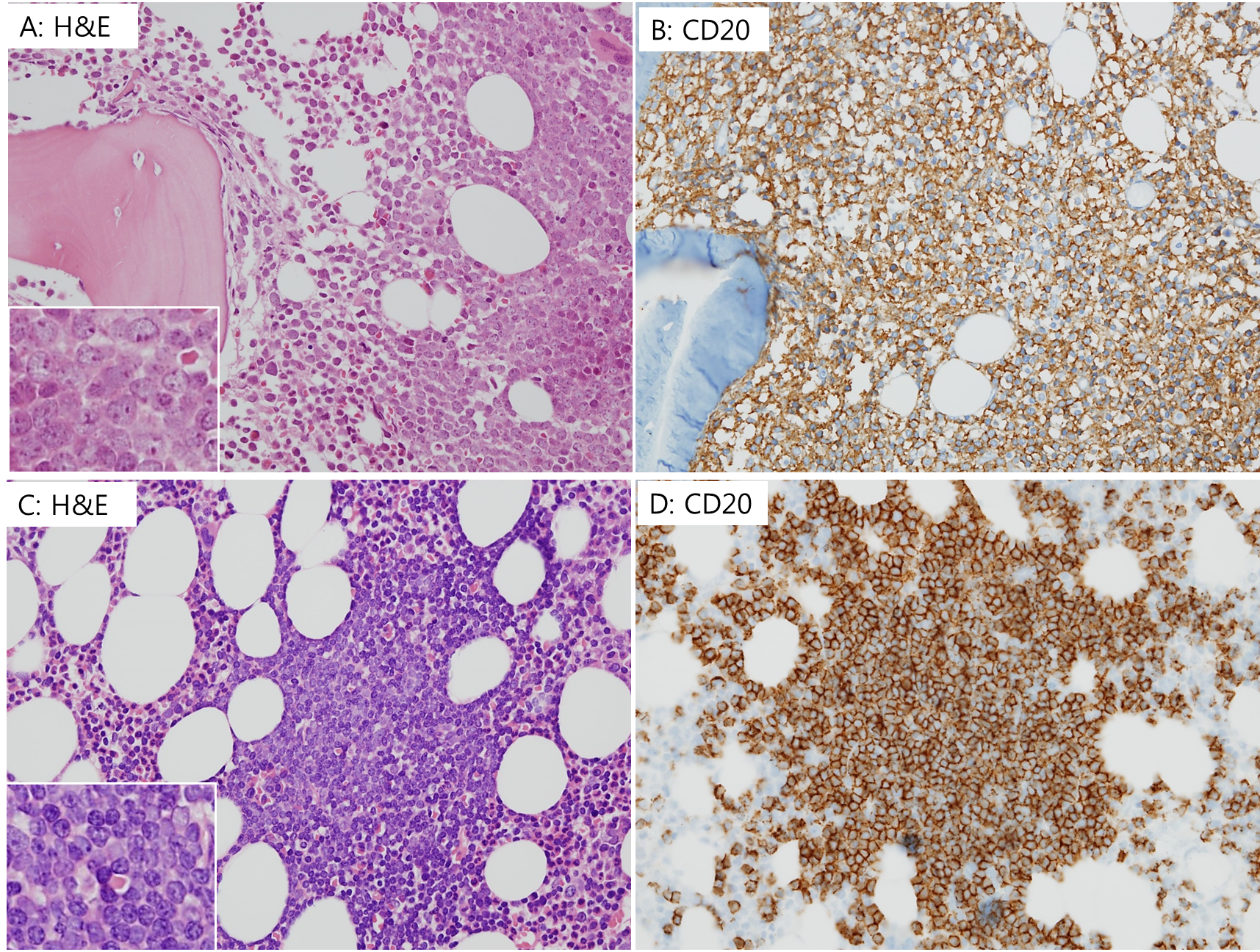

Supplement: Supplementary file 1 — Figure S1. (A) A representative case of the involvement of large B‐cell lymphoma (DLBCL) that shows diffuse proliferation of large B cells (A and B). A representative case of involvement of small cell B‐cell lymphoma that shows small‐ to medium‐sized low‐grade mature B cells in patterns of large aggregates of infiltrative edges, occupying large proportions of marrow surfaces (C and D). Representative cases of small nonparatrabecular, interstitial aggregates of small‐ to medium‐sized mature B cells, which did not fulfill the criteria of lymphoma due to low cellularity of the proliferating B cells or lack of histologic characteristics for malignant lymphoid aggregates (E–F and G–H). Figures of H&E staining and CD20 immunostaining were captured at ×200 magnification. Figures of insets show more details of tumor cells. [file CAM4-5-1066-s001.tif]

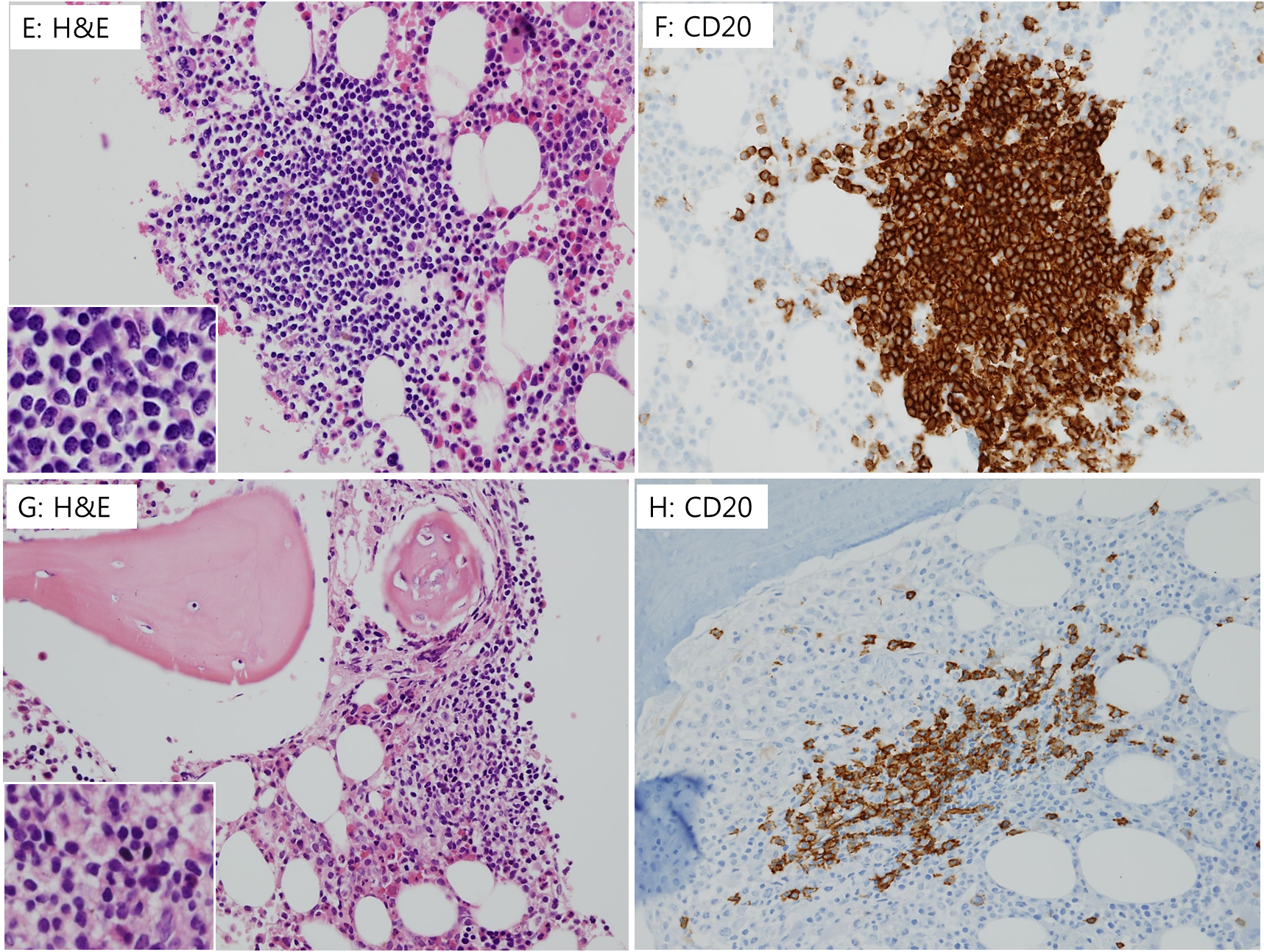

Supplement: Supplementary file 2 [file CAM4-5-1066-s002.tif]
